# Non-Genomic AhR-Signaling Modulates the Immune Response in Endotoxin-Activated Macrophages After Activation by the Environmental Stressor BaP

**DOI:** 10.3389/fimmu.2021.620270

**Published:** 2021-03-31

**Authors:** Henning Großkopf, Katharina Walter, Isabel Karkossa, Martin von Bergen, Kristin Schubert

**Affiliations:** ^1^ Department of Molecular Systems Biology, Helmholtz Centre for Environmental Research, Leipzig, Germany; ^2^ Institute of Biochemistry, Leipzig University, Leipzig, Germany

**Keywords:** aryl hydrocarbon receptor, macrophages, proteome, ubiquitin, phosphorylation, immunomodulation, Rac1, AKT

## Abstract

Emerging studies revealed that the Aryl hydrocarbon receptor (AhR), a receptor sensing environmental contaminants, is executing an immunomodulatory function. However, it is an open question to which extent this is achieved by its role as a transcription factor or *via* non-genomic signaling. We utilized a multi-post-translational modification-omics approach to examine non-genomic AhR-signaling after activation with endogenous (FICZ) or exogenous (BaP) ligand in endotoxin-activated (LPS) monocyte-derived macrophages. While AhR activation affected abundances of few proteins, regulation of ubiquitination and phosphorylation were highly pronounced. Although the number and strength of effects depended on the applied AhR-ligand, both ligands increased ubiquitination of Rac1, which participates in PI3K/AKT-pathway-dependent macrophage activation, resulting in a pro-inflammatory phenotype. In contrast, co-treatment with ligand and LPS revealed a decreased AKT activity mediating an anti-inflammatory effect. Thus, our data show an immunomodulatory effect of AhR activation through a Rac1ubiquitination-dependent mechanism that attenuated AKT-signaling, resulting in a mitigated inflammatory response.

## Introduction

The Aryl hydrocarbon receptor (AhR), initially described as an environmental sensor (1), has been shown to play a pivotal role in the modulation of immune cell function (2). Most immune cells, including macrophages, and their progenitors, express the AhR (3–5). On the one hand, AhR can inhibit the differentiation of monocyte-derived and bone marrow-derived macrophages (6, 7). On the other hand, AhR decreases the production of proinflammatory cytokines and increases the secretion of anti-inflammatory IL-10 after pattern recognition receptor (PRR) activation by differentiated macrophages (8). Macrophages of AhR-knockout (Ahr^-/-^) mice produce higher amounts of proinflammatory cytokines (TNF-α, IL-6, IL-12). These AhR^-/–^mice are more sensitive to LPS induced septic shock (9, 10), suggesting an anti-inflammatory effect protecting against immunopathologies (11). However, the underlying molecular mechanisms are not entirely understood.

The AhR is a ligand-activated transcription factor and was first described as a receptor for 2,3,7,8-tetrachlorodibenzo-p-dioxin (TCDD) (1). Additional exogenous chemicals like benzo(a)pyrene (BaP) but also endogenous ligands like the light-dependent Tryptophan metabolite 6-formylindolo[3,2-b]carbazole (FICZ) and metabolites produced by the intestinal microbiota have been shown to activate the AhR (12, 13). AhR is part of a cytosolic complex in its inactive form from which AhR is released upon ligand binding, resulting in AhR translocation to the nucleus (14–17). After dimerization with the aryl hydrocarbon receptor nuclear translocator (ARNT), gene expression from xenobiotic response elements (XRE) is initiated (18). Among the target genes are phase I xenobiotic-metabolizing enzymes, e.g., CYP1A1, CYP1B1, IDO1, and TDO2, as well as the aryl hydrocarbon receptor repressor (AHRR), providing a negative feedback loop (19).

Besides its function as a transcription factor, AhR has been shown to exhibit non-genomic signaling pathways by regulating protein ubiquitination and phosphorylation. The AhR itself can act ligand-dependently as the substrate recognition unit of a cullin 4B ubiquitin ligase complex targeting the estrogen receptor α (ERα) for proteasomal degradation (20). Thereby, the control of AhR localization is a regulatory mechanism to modulate transcriptional activity and ubiquitin ligase function (21). On the other hand, AhR ligand binding can release the tyrosine-protein kinase SRC from the cytosolic AhR-complex in an active form (14), resulting in phosphorylation of the E3 ubiquitin ligase c-CBL and subsequent ubiquitination and degradation of the spleen tyrosine kinase (SYK) in pre-osteoclast after AhR activation with tetrandrine (22).

Both genomic and non-genomic signaling pathways contribute to the AhR-dependent modulation of macrophage activation. Two of the NF-κB subunits (RelA and RelB) can physically interact with AhR (23, 24). AhR activation by TCDD induces degradation of RelA by the ubiquitin-proteasome pathway. This effect is dependent on AhR transcriptional activity (25). Zhu et al. could demonstrate that non-genomic AhR-signaling participates in immunomodulation through SRC-dependent STAT3 phosphorylation and subsequent anti-inflammatory IL-10 production (26), revealing its importance in immune function. However, although knowledge on non-genomic AhR-signaling is emerging, little is known on the underlying molecular mechanisms in macrophages.

Thus, we aimed to unravel the immunomodulatory properties and the relevant non-genomic signaling events after AhR activation by BaP or FICZ using a multi-PTM-omics approach in endotoxin-stimulated human monocyte-derived macrophages. We analyzed alterations of protein ubiquitination and phosphorylation to elucidate affected signaling pathways after AhR activation and integrated the data to characterize the contribution of non-genomic AhR-signaling in macrophages.

## Material and Methods

### Cell Culture

Buffy coats were obtained from healthy donors from the Institute of Transfusion Medicine, Leipzig, Germany as approved by the local ethic committee. Peripheral blood mononuclear cells (PBMC) were enriched from buffy coats by Ficoll gradient centrifugation. Primary monocytes were enriched from PBMC by adherence to the surface of cell culture plates for 2h in RPMI 1640 (Life Technologies, USA) supplemented with 5% heat-inactivated FBS (Biowest, France) and Penicillin/Streptomycin (Sigma-Aldrich, USA). Monocytes were collected and differentiated to macrophages by adding 100ng/ml recombinant human M-CSF (PeproTech, Germany) for 5d, as described previously (27). Differentiated macrophages were collected and distributed to new cell culture plates and rested for 24h. Rested macrophages were stimulated with 2µM BaP (Sigma-Aldrich, USA), 100nM FICZ (Enzo Biochem, USA), or the same volume DMSO (Sigma-Aldrich, USA) for 2h. All stimulations were additionally conducted with 100ng/ml LPS (InvivoGen), USA). Cells were treated with 2µM Perifosine (Cell Signaling Technologies, USA) or 25µM RAC1-Inhibitor NSC23766 (ENZ-CHM116, Enzo Life Sciences, USA) 30min before stimulation with AhR-ligands and/or LPS for inhibitor assays. All cell culturing incubations were conducted in a humidified cell incubator at 37°C with 5% CO_2_. The cells were lysed with urea lysis buffer (9M urea (Merck, Germany) in 20mM HEPES pH 8.0 (Sigma-Aldrich, USA) supplemented with 1mM sodium orthovanadate (Sigma-Aldrich, USA), 2.5mM sodium pyrophosphate (Sigma-Aldrich, USA), and 1mM b-glycerol-phosphate (Alfa Aesar, USA)) for proteome, ubiquitome, and phosphoproteome analysis. Cell lysis for western blot analysis was conducted with Triton X-100 lysis buffer (1% Triton-X-100 (SERVA, Germany), 150mM NaCl (Roth, Germany), 0.5% Na-deoxycholate (Sigma, USA), 0.5% SDS (SERVA, Germany) in 50mM TrisHCl pH 7.4 (Merck, Germany)).

### Fractionation and Digestion for LC-MS/MS-Based Proteomics

The protein concentrations of the samples were determined utilizing the Pierce 660 nm proteins assay (Thermo Fisher, USA). For global proteome analysis, 30 µg protein were pre-fractionated by SDS-PAGE as previously described (28). Each sample lane was cut in 5 slices with approximately the same protein amount after Coomassie staining. All fractions were reduced with 100 mM DTT (Merck, Germany), carbamidomethylated with 100 mM IAA (Merck, Germany), and proteolytically cleaved in-gel utilizing Trypsin (Roche, Switzerland) applied in a 1:40 Trypsin/protein ratio. The resulting peptides were extracted with 50% ACN in 0.01% FA. Peptide extracts were evaporated with a vacuum-concentrator and reconstituted in 0.1% FA for LC-MS/MS measurement. Seven biological replicates (n = 7) were used for proteome analysis.

### Ubiquitin-Remnant-Motif (K-ε-GG) Analysis

Ubiquitome analysis was conducted by PTMScan^®^ Discovery Proteomic Services (Cell Signaling Technology, Danvers, MA, USA). In short, pooled lysates from nine donors were digested using Trypsin, and peptides with the ubiquitin-remnant-motif (K-ϵ-GG-remnant), which is left on ubiquitinated lysine residues after tryptic digestion, were enriched utilizing a proprietary antibody. All Ub-remnant enriched samples were measured in technical duplicates by LC-MS/MS.

### Phosphopeptide Enrichment

For phosphopeptide enrichment, samples were proteolytically cleaved using a paramagnetic bead approach (29, 30). In short, 600µg protein of each sample was precipitated on Magnetic Carboxylate Modified Particles (Sigma-Aldrich, USA), reduced with 50 mM TCEP (Sigma-Aldrich, USA), carbamidomethylated with 100 mM IAA (Merck, Germany), and proteolytically cleaved with Trypsin (Promega, USA) applied in a 1:50 Trypsin/protein ratio. Peptides were collected and dried to completeness using a vacuum concentrator.

Phosphopeptides were sequentially enriched by TiO2- and Fe-NTA-based affinity chromatography. Firstly, the High-Select™ TiO_2_ Phosphopeptide Enrichment Kit (Thermo Scientific, USA) was used following the manufacturer’s instructions. Flow-through of peptide samples and first wash fractions were combined and dried to completeness for the following second enrichment step. Secondly, the High-Select™ Fe-NTA Phosphopeptide Enrichment Kit (Thermo Scientific, USA) was used following the manufacturer’s instructions. Phosphopeptide enriched eluates from both steps were combined, dried to completeness, and reconstituted in 0.1 % FA for LC-MS/MS measurement. Four biological replicates (n = 4) were conducted for Phosphopeptide enrichment.

### LS-MS/MS

LC-MS/MS analysis of samples was performed on an UltiMate 3000 RSLCnano system (Dionex, USA), online coupled to a Q Exactive HF mass spectrometer (Thermo Fisher Scientific, USA) by a chip-based electrospray ionization source (TriVersa NanoMate, Advion, USA). Peptides were trapped and desalinated on a C18 pre-column (Acclaim PepMap 100, 75 μm x 2 cm, C18, 3 μm), and subsequently separated on a C18 analytical column (Acclaim PepMap RSLC, 75 μm x 25 cm, C18, 2 μm).

For proteome analysis, a previously described bipartite linear 55 min gradient starting from 4 % eluent B (0.1 % FA in 80 % ACN) in eluent A (0.1 % FA in water) to 55 % eluent B *via* 30 % eluent B after 47.5 min was used (28). After each sample, the column was flushed to 99% eluent B and reconstituted to starting conditions. Mass spectra were acquired in a data-dependent manner. For MS1 scans, the following parameters were set: m/z range 350-1550, maximum injection time = 100 ms, automated gain control (AGC) = 3x10^6^, R = 60 000. The top 10 most abundant ions were selected for MS2 acquisition using the following parameters: isolation window of 1.4 m/z, maximum injection time 100 ms, AGC = 2x10^5^, normalized collision energy (NCE) = 28, R = 15 000, m/z range 200-2000. Fragmented ions were dynamically excluded for 20 s.

For phosphopeptide analysis, a tripartite linear 145 min gradient starting from 4 % eluent B (0.1 % FA in 80 % ACN) in eluent A (0.1 % FA in water) to 55 % eluent B *via* 18 % eluent B after 77.5 min and 30 % eluent B after 115 min was used. After each sample, the column was flushed to 99% eluent B and reconstituted to starting conditions. Mass spectra were acquired in a data-dependent manner. For MS1 scans the following parameters were set: m/z range 350-1550, maximum injection time = 120 ms, AGC = 3x10^6^, R = 120 000. The top 15 most abundant ions were selected for MS2 acquisition using the following parameters: isolation window of 0.7 m/z, maximum injection time 150 ms, AGC = 2x10^5^, normalized collision energy (NCE) = 28, R = 15 000, m/z range 200-2000. Fragmented ions were dynamically excluded for 45 s.

### Data Analysis

The LC-MS/MS raw data for proteome and phosphoproteome were examined by MaxQuant (Version 1.6.7.0) (31). Database search was performed against the Uniprot Homo Sapiens RefSet (09/2019, 74349 entries) and a list of common contaminants provided by MaxQuant (07/2019, 245 entries) (32). Search parameters were set as follows: Maximum missed cleavages = 2, minimal peptide length = 6 amino acids, first search peptide tolerance = 20 ppm, main search peptide tolerance = 4.5 ppm, FTMS MS/MS match tolerance = 20 ppm. Carbamidomethylation of cysteine was set as fixed modification, protein N-terminal acetylation, oxidation of methionine, and, for phosphopeptide enriched samples, phosphorylation of Serin, Threonine, and Tyrosine were set as variable modifications. Peptides, proteins, and sites were filtered by a target-decoy approach to an FDR ≤ 0.01 using a reversed decoy database. Match between runs was enabled with a match time window of 0.7 min and alignment time window of 20 min. Label-free quantification (LFQ) was used for relative protein quantification based on an LFQ ratio count ≥2.

Proteins and phosphosites identified by site, from the reverse database, or as potential contaminants were removed. R-3.6.1 was used for further statistical analysis using the following packages: limma (33), plyr (34), reshape2 (35), xlsx (36), DEP (37), ggsci (38), circlize (39), calibrate (40), ggplot2 (41), readxl (42), qpcR (43), splitstackshape (44), tidyr (45), and Tmisc (46). (LFQ-) intensities were log2-transformed and median normalized. To be considered as reliably quantified, proteins or PP-sites had to be quantified in more than 50% of replicates. Imputation was done for proteins and PP-sites completely not quantified in one condition but reliably quantified in the second condition of the comparison. Significantly altered proteins and PP-sites were then identified by Student’s t-test. The test was chosen because of its applicability to the used quantification method, number of biological replicates, and statistical power (47). Using this test, false positives are expected evenly distributed among all quantified proteins and phosphosites, while true changes cluster in relevant altered pathways. Hence, pathway- and enrichment-based analysis provide an additional filter (48). The down-stream analysis was largely based on enrichment analyses for pathways and inference of kinase activities. The threshold for significant alteration was set at p ≤ 0.01 without FDR correction to evade double filtering but limit the number of by chance false positives.

Functional annotation regarding KEGG and Reactome pathways was done with DAVID bioinformatics resources (49). Ingenuity pathway analysis (IPA) was used for phosphorylation-dependent pathway analysis. Molecules and relationships were restricted the following: species = human, confidence = experimentally observed, tissues = monocyte-derived macrophages. Only pathways with a p-value ≤ 0.01 were used for further analyses. Kinase activities were inferred by integrating amino acid sequence windows of significantly altered PP-sites with kinase-substrate motifs utilizing KinSwingR (50) in the R environment. Kinase substrate motifs were calculated based on the kinase-substrate dataset from PhosphoSitePlus (downloaded 12/2019) (51).

### Enzyme-Linked Immunosorbent Assay (ELISA)

Supernatants of monocyte derived macrophages were collected after stimulation and analyzed for TNF, IL1β and NO release. The BD OptEIA™ Human TNF ELISA Set(BD Biosciences, USA), BD OptEIA™ Human IL1β ELISA Set (BD Biosciences, USA), and Total Nitric Oxide and Nitrate/Nitrite Parameter Assay Kit (Bio-Techne, Germany) were applied according to the manufacturer’s protocol.

### Quantification of mRNA Expression Levels

QTotal RNA was isolated using the RNeasy kit (QIAGEN, Germany), and single stranded cDNA was synthesized using the High Capacity cDNA Reverse Transcription Kit (Thermo Fisher, USA). The qPCR was performed using TaqMan™ Fast Advanced Master Mix (Thermo Fisher, USA) and a qPCR ABI 7500 Fast Real-Time PCR System (Applied Biosystems, USA). The following primers were used 1) Hs00164383_m1, CYP1B1, FAM-MGB 2) Hs00174128_m1, TNFalpha, FAM-MGB 3) Hs00420895_gH, RPLPO, FAM-MGB 4) Hs01005075_m1, AHRR, FAM-MGB 5) Hs01054794_m1, CYP1A1, FAM-MGB (all Thermo Fisher, USA). Fold changes were calculated by the ΔΔc_t_-Methode relative to the unstimulated control with RPLP0 as the reference (52).

### Western Blot Analysis

Cell lysates were separated by SDS-PAGE (10%), and transferred onto a nitrocellulose membrane. Membranes were blocked in 3% BSA in TBS-T (0.05% Tween20 in Tris-buffered saline) for 1h, followed by incubation with primary antibody overnight at 5°C. The following primary antibodies were used: β-Actin (#3700, Cell Signaling Technologies, USA), phospho-AKT Ser473 (#9271, Cell Signaling Technologies, USA), AKT (#9272, Cell Signaling Technologies, USA), AhR (sc-133088, Santa Cruz Biotechnology, USA) and CDKN1A (#2947, Cell Signaling Technologies, USA). Immunoblots were washed with TBS-T, and incubated with HRP-coupled secondary anti-rabbit (#1706515, Biorad, USA) or anti-mouse (#7076, Cell Signaling Technologies, USA) antibody for 1h at room temperature. After washing 3-times with TBS-T, chemiluminescence signal was detected using the FluorChem FC3 imager (ProteinSimple, USA). Quantification was performed using AlphaView SA (Version 3.5.0.927, ProteinSimple, USA).

## Results

### Effect of AhR-Ligands on Target Gene Expression and TNF-Release

**Figure 1:**
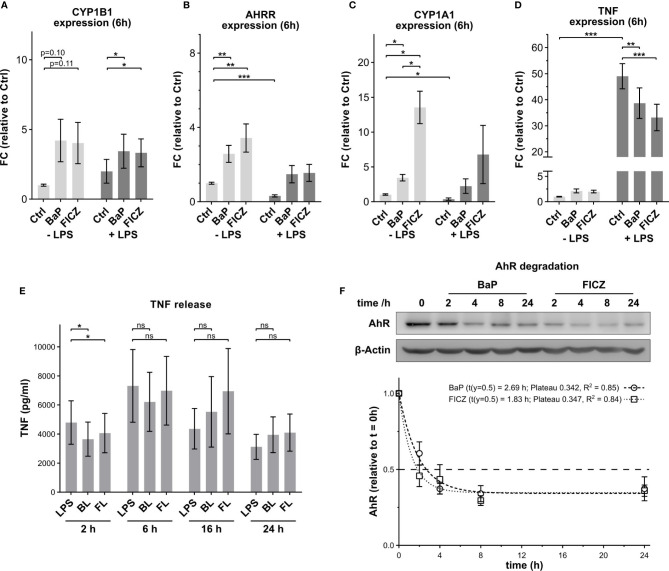
Effect of AhR-ligands on target gene expression and TNF-release. The mRNA expression levels of the AhR-target genes CYP1B1 **(A)**, AHRR **(B)**, and CYP1A1 **(C)** were determined by qPCR after 6h stimulation with 2 µM BaP or 100 nM FICZ. **(D)** The TNF mRNA expression levels were determined by qPCR 6h post stimulation with 2 µM BaP or 100 nM FICZ in the absence and presence of 100 ng/ml LPS. Data were normalized to RPLP0, and the gene expression levels were calculated relative to the unstimulated control. Data are shown as mean ± SEM (n = 5). **(E)** TNF release in cell supernatant. TNF concentrations in the supernatants were measured by ELISA 2h, 6h, 16h, and 24h after treatment. Data are shown as mean ± SEM (n = 5). Significant changes were determined by two-sided, paired t-test (***p ≤ 0.001, **p ≤ 0.01, *p ≤ 0.05; ns, not significant). **(F)** The degradation of AhR in response to 2 µM BaP or 100 nM FICZ was determined by western blot. AhR signal intensities were normalized to β-Actin levels. Shown is one representative blot and normalized intensities relative to t = 0h ± SEM (n = 4). The degradation was analyzed as one phase decay by least squares fit.

To study the immunomodulatory properties of non-genomic AhR-signaling in macrophages, we investigated the effects of an endogenous (FICZ) and an exogenous (BaP) AhR ligand. The effects of BaP and FICZ on AhR-target gene expression were evaluated in human monocyte derived-macrophages to confirm comparable capacities of AhR activation. After 6h of treatment, both ligands led to a ~4-times induction of CYP1B1 mRNA expression (**Figure 1A**) at the used concentrations of 2 µM (BaP) and 100 nM (FICZ). The AhR repressor (AHRR) was increased 2.8-times by BaP and 3.2-times by FICZ (**Figure 1B**). In contrast, the induction of CYP1A1 expression was about 3-times stronger after FICZ-treatment, than after BaP-treatment. Co-treatment with either of the two ligands and 100 ng/ml LPS attenuated the AhR-ligand dependent induction of AHRR and CYP1A1, but not CYP1B1 (**Figures 1A–C**). The stimulation of macrophages with LPS strongly induced TNF mRNA expression by a factor of ~50. Co-treatment with the AhR-ligands BaP and FICZ inhibited this induction by 21% and 32%, respectively, while AhR activation alone led to a slight increase of TNF-mRNA levels (**Figure 1D**). Thus, BaP and FICZ activated AhR to a comparable extent at the given concentrations, and both ligands had an inhibiting effect on TNF expression after LPS-treatment.

To evaluate the effects of AhR activation, macrophages were treated for 2h, 6h, 16h and 24h with the respective AhR-ligands with and without LPS. After 2h, the AhR activation by BaP or FICZ in combination with LPS, subsequently termed BL and FL, mitigated the LPS-induced TNF-release indicating a modulatory effect (**Figure 1E**). BaP and FICZ reduced the LPS-dependent TNF-release by 23% and 17%, respectively, while the AhR-ligands did not affect the TNF-release without LPS-co-stimulation (**Supplementary Figure 1A**). With longer incubation time, the repression of TNF-release by the AhR-ligands diminished after 6h. Neither LPS-treatment, nor treatment with AhR-ligands alone or in combination with LPS- induced release of nitric oxide after 2h (**Supplementary Figure 1B**). Additionally, co-treatment with LPS and AhR-ligand had no significant effect on the release of IL1β compared to LPS alone (**Supplementary Figure 1C**). The IL1β levels in the supernatant were minute after 2h and increased over the complete observation time. Beginning after 16h, AhR-stimulation showed an augmenting effect on the LPS-induced IL1β release, although this effect was not statistically significant.

Previous reports showed that AhR is rapidly degraded after activation in hepatocytes. Thereby, nuclear export of AhR precedes the degradation by the 26S-proteasome with a half-life of 2–3h (53, 54). The speed of AhR-degradation in macrophages was determined by western blot analysis (**Figure 1F**). Both treatments, with 2µM BaP or 100nM FICZ, led to AhR-degradation. Half of the AhR amount was degraded after 2.7h and 1.8h for BaP and FICZ, respectively. Notably, one-third of AhR was not degraded for both ligands within the investigated time frame, suggesting that this AhR fraction does not enter the nucleus and is subject to nuclear export and subsequent degradation.

It was previously shown, that most changes in protein abundances can be found 4h after AhR activation and earlier time points show few altered proteins (55), thus minimizing the potential impact of newly synthesized proteins on post-translational modification. Hence, 2h treatments were used to investigate the participation of AhR-dependent post-translational modification in immunomodulation. After stimulation, cells were lysed, and the total proteome, ubiquitome, and phosphoproteome were analyzed utilizing label-free quantitative LC-MS/MS.

### Proteomic Changes Induced by AhR-Ligands

First, the effects of AhR activation on the global proteome were investigated in endotoxin-stimulated macrophages. Alteration of protein levels after stimulation with AhR-ligand with and without LPS was investigated for seven donors, resulting in 5327 identified proteins. A reliably quantified core proteome, meaning quantified in at least four replicates of one treatment, of 4336 proteins was defined for further analysis (**Supplementary Table 1**).

**Figure 2:**
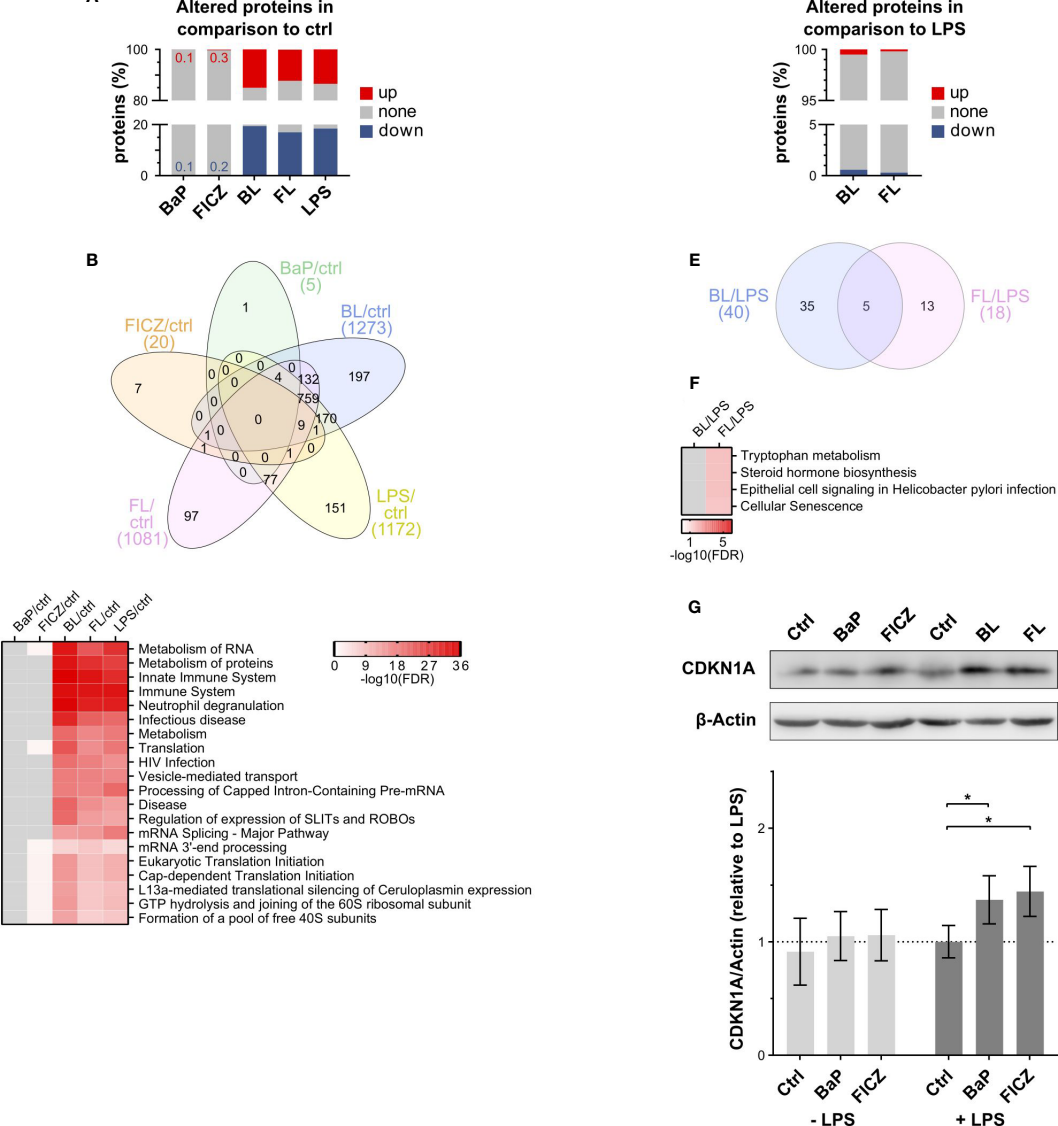
Proteomic analysis of AhR-ligand treated macrophages in the presence/absence of LPS. **(A)** Percentage of significantly altered proteins after 2h compared to DMSO (ctrl). Proteins were considered as significantly altered compared to the control with a p-value ≤ 0.01, as determined by Student’s t-test (n = 7). **(B)** Overlap of altered proteins between treatments. **(C)** Pathways enriched among regulated proteins compared to the unstimulated control. Pathway enrichment analysis was conducted using the DAVID bioinformatics resources against KEGG and Reactome reference databases. Shown are the top 10 pathways based on their false discovery rate (FDR) for each treatment. **(D)** Percentage of significantly altered proteins in ligand- and LPS-stimulated cells in comparison to LPS alone (p-value ≤ 0.01, n = 7). **(E)** Overlap of proteins altered by AhR-ligands under inflammatory conditions. **(F)** Pathways enriched among regulated proteins in BL- and FL-treated cells compared to LPS-treatment. Shown are all significantly enriched pathways (FDR ≤ 0.05). (BL: BaP+LPS-treated; FL: FICZ+LPS-treated) **(G)** Relative CDKN1A protein levels were determined by western blot analysis. CDKN1A signal intensities were normalized to β-Actin. Shown is one representative blot out of 6 independent experiments and donors and normalized intensities relative to LPS-treatment ± SEM (n = 6). Significant changes were determined by two-sided, paired t-test (*p ≤ 0.05).

The stimulation with AhR-ligands led to a minimal number of significantly altered proteins (p ≤ 0.01) after 2h (**Figure 2A**). BaP-treatment affected the abundance of five proteins. Three of them were up-regulated compared to the unstimulated control, and two were down-regulated. FICZ-treatment led to the down-regulation of 12 proteins and up-regulated eight. In contrast, LPS had a strong effect on the proteome. Roughly one-third of proteins were significantly affected after LPS, BL, and FL treatment (**Supplementary Figure 2**). At that, more proteins were down-regulated (18.5%) than up-regulated (13.5%) after LPS-treatment. A similar distribution was observed for BL (19.4% up, 15% down) and FL (17% up, 12.2% down). Interestingly, proteins regulated by stimulation with BaP and FICZ did not show any overlap, while most proteins were commonly regulated after stimulation with LPS, BL, and FL (**Figure 2B**). A total of 1251 proteins was altered under at least two of these conditions, with 772 being commonly regulated for all treatments. Next, pathway enrichment analysis was conducted to identify cellular processes affected by protein regulations. For LPS, FL, and BL-treatment, metabolism of RNA and proteins were most affected, reflecting the high number of regulated proteins (**Figure 2C**). Furthermore, processes of the (innate) immune response and ongoing infectious diseases were regulated (**Figure 2C**). The pathways enriched after FICZ treatment were mainly related to mRNA-translation. Due to the limited number of regulated proteins, no pathways were enriched in BaP-treated cells.

To analyze the immunomodulatory function of AhR under inflammatory conditions in more detail, the double-stimulated samples, BL and FL, were additionally compared to LPS-treated cells (**Figure 2D**). Although the number of differentially abundant proteins was small, five proteins were commonly affected by BL and FL compared to LPS alone (**Figure 2E**). The levels of SLAM family member 8 (SLAMF8) and Disintegrin and metalloproteinase domain-containing protein 17 (ADAM17) were decreased, whereas the levels of Ubiquilin-1 (UBQLN1), Cyclin-dependent kinase inhibitor 1 (CDKN1A), and Cytochrome P450 1B1 (CYP1B1) were increased (**Supplementary Figure 3**). Pathway enrichment analysis resulted in no significantly enriched pathways within the differentially abundant proteins comparing BL and LPS (**Figure 2F**). Proteins participating in the Tryptophan metabolism and Steroid hormone biosynthesis were enriched comparing FL and LPS-treated cells (**Figure 2F**). BaP metabolites can induce DNA damage through formation of adducts (56). Hence, the increase of CDKN1A levels, which is an inhibitor of cell cycle progression in response to DNA damage (57) and blocks M-CSF dependent macrophage proliferation in response to IL4 (58), was confirmed by western blot analysis (**Figure 2G**). Both, BL and FL, showed significantly increased CDKN1A signals compared to LPS, suggesting a potential inhibition of macrophage *in situ* proliferation.

Taken together, AhR activation by BaP or FICZ had a limited effect on protein abundances after 2h. In contrast, LPS-stimulation regulated many proteins involved in RNA and protein metabolism and innate immune responses, reflecting the increased gene expression at the early activation status (59). However, the effects of co-stimulation with AhR-ligands were similarly limited as for stimulation with BaP or FICZ alone, suggesting only a minor role of AhR activation on the macrophage proteome after 2h. Hence, non-genomic signaling by AhR might be responsible for the observed reduction of TNF expression and release.

### AhR-Stimulation Affects Ubiquitination of Multiple Proteins in Macrophages

Activation of AhR has been shown to alter ubiquitination and proteasomal degradation of proteins (20). Thus, we aimed to characterize the ubiquitination state of cellular proteins after AhR-stimulation by BaP or FICZ with and without LPS- treatment. Lysates of stimulated macrophages from nine healthy donors were pooled, digested, and peptides harboring the Ubiquitin-Remnant-Motif (K-ε-GG) were enriched and quantified by LC-MS/MS in duplicates.

**Figure 3:**
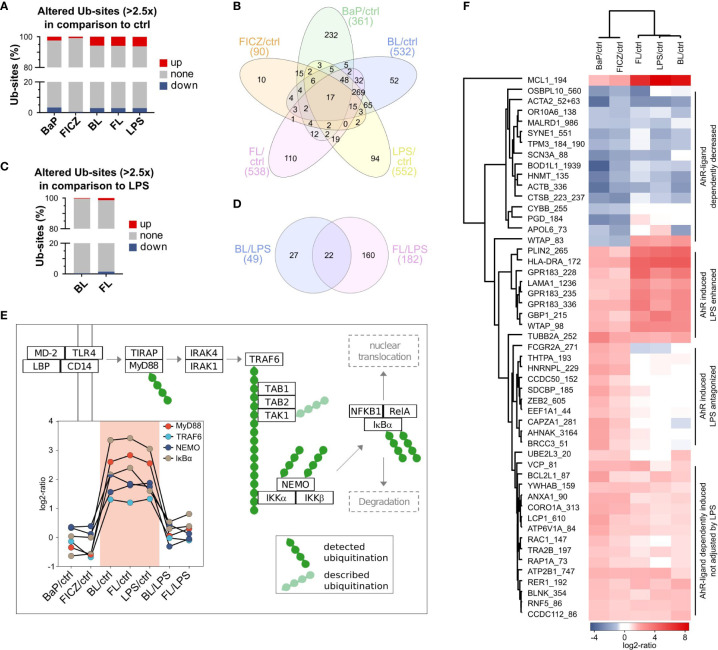
Effect of AhR activation on protein ubiquitination **(A)** Rates of ubiquitination sites (Ub-sites) with altered abundance after 2h in comparison to untreated control. Ub-sites with a FC ≥ 2.5 and a maximal CV ≤ 50% were considered as differentially abundant. Ubiquitome samples were pooled from 9 donors and measured in duplicates. **(B)** Overlap of altered Ub-sites between different treatments. **(C)** Rates of altered Ub-sites in double-treated macrophages in comparison to LPS-treatment. **(D)** Overlap of altered Ub-sites by AhR-ligands under inflammatory conditions. **(E)** Regulation of ubiquitination in MyD88-dependent TLR4 signaling. Obtained ubiquitome data were searched for proteins described as ubiquitinated after TLR4-activation. Detected Ub-sites are shown in dark-green and described but not detected Ub-sites in pale-green. **(F)** Ub-sites mutually regulated by BaP and FICZ treatment.

In total, 5,975 ubiquitination-sites (Ub-sites) were quantified, and Ub-sites with a fold change ≥2.5 relative to the respective control were considered as altered (**Supplementary Table 2**) (**Supplementary Figure 4**). As for proteins, most alterations occurred in samples treated with LPS, regardless of co-stimulation with AhR-ligands (**Figure 3A**). Thereby, twice as many Ub-sites were increased than reduced in abundance in LPS-treated samples, whereas such a difference was not observed after treatment with AhR-ligands alone (**Figure 3A**).

Most of the 90 Ub-sites altered by FICZ-treatment were affected by BaP, BL, or FL as well. Only ten sites were unaffected by all other treatments (**Figure 3B**). In contrast, 64% of Ub-sites altered by BaP-treatment were specific to this treatment. The overlap of altered Ub-sites after stimulation with LPS, BL, and FL was similar to the overlap of regulated proteins. A total of 490 Ub-sites was altered under at least two of these conditions, with 269 commonly regulated for all treatments (**Figure 3B**). About 10 to 20% of the Ub-site-alterations under one condition were unique for this treatment.

Comparing ubiquitination after double-stimulation to LPS-treated cells, FICZ affected more than 3-times more Ub-sites than BaP (**Figure 3C**). Thereby, increase and decrease of abundance occurred at the same frequency for both ligands. 22 Ub-sites were commonly altered for BL and FL compared to LPS (**Figure 3D**).

Stimulation of macrophages with LPS leads to activation of the Toll-like receptor 4 (TLR4) (60). Activation of TLR4 has been shown to trigger three signaling axes: The PI3K-Akt signaling, the MyD88-dependent, and the MyD88-independent pathway. As part of the MyD88 dependent signaling cascade, the ubiquitination of multiple proteins is essential for the signaling cascade (61). In our experiments, MyD88, TRAF6, NEMO, and IκBα were found ubiquitinated, with NEMO and IκBα being ubiquitinated at two sites (**Figure 3E**). All six Ub-sites were up-regulated upon LPS-treatment. The strongest increase was found for the ubiquitination of IκBα at Lysine 98 (IκBα_98), which was more than 8-times increased. This up-regulation was independent of co-stimulation with BaP or FICZ. IκBα_238 exhibited the highest difference between double-stimulated (BL or FL) and LPS-treated cells (1.75x FL/LPS and 1.45x BL/LPS). Thus, AhR activation did not interfere with ubiquitination in MyD88-dependent TLR4-signaling.

Next, we asked which ubiquitinations were concurrently altered after AhR-stimulation. Therefore, we filtered for Ub-sites, which were consistently ≥2.5-times more or less abundant in BaP and FICZ-treated samples than in the unstimulated control. Of 51 mutually affected Ub-sites in 48 proteins, 36 Ub-sites were more abundant in BaP- and FICZ-treated macrophages as in unstimulated control cells, whereas 15 Ub-sites had a decreased abundance. Cluster analysis of these Ub-sites revealed three distinct groups of AhR-ligand dependently induced ubiquitination concerning their response to LPS: (1) induction not affected by LPS, (2) antagonized by LPS, and (3) LPS-enhanced induction (**Figure 3F**). However, such clustering was not observed for AhR-ligand dependently reduced Ub-sites. While LPS induced most of the Ub-sites in group 1 to a similar degree, ubiquitination of Ras-related protein Rac1 (RAC1_147) and Ras-related protein Rap-1A (RAP1A_73) was not induced by LPS alone. Thus, the Ub-sites RAC1_147 and RAP1A_73 were the only sites induced by both AhR-ligands completely independent of LPS. Interestingly, both proteins exhibit GTPase-activity (62), indicating modulation of small GTPase-activity as a potential mechanism of non-genomic AhR-signaling.

Pathway enrichment analysis for the set of proteins ubiquitinated mutually regulated was conducted utilizing DAVID against the KEGG pathway database. Four pathways were significantly enriched (FDR ≤ 0.05): Phagosome, Osteoclast differentiation, Viral myocarditis, and PI3K-Akt signaling pathway (**Supplementary Figure 5**). With seven proteins (ACTB, ATP6V1A, CORO1A, TUBB2A, RAC1, FCGR2A, HLA-DRA), the highest number of proteins participates in phagosomal processes. Five proteins (LAMA1, MCL1, RAC1, YWHAB, BCL2L1) were associated with the PI3K-Akt signaling pathway, one of the signaling axes of activated TLR4.

Our ubiquitome data showed that AhR-activation did not interfere with Ub-dependent processes in MyD88-dependent TLR4-signaling. LPS-treatment dominated most regulations of Ub-sites. However, with RAP1A_73 and RAC1_147, two Ub-sites in GTPases were AhR-ligand-dependently, and LPS-independently induced. Both proteins participate in phagocytosis, and RAC1 participates in PI3K-Akt signaling as well. These two pathways were enriched among differentially ubiquitinated proteins after AhR activation, indicating a regulatory role of the RAC1-PI3K-AKT signaling pathway and affected phagocytosis.

### Effect of AhR-Stimulation on Protein Phosphorylation Is Ligand-Specific

Many biological processes, including the RAC1-PI3K-AKT signaling pathway, are regulated by protein phosphorylation, which influences protein activity. AhR activation can affect protein-phosphorylation, e.g., by releasing SRC from the cytoplasmic AhR-complex (14). Thus, the phosphorylation state of cellular proteins was analyzed next. Again, macrophages were stimulated with BaP or FICZ alone or in combination with LPS for 2h. Phosphorylated peptides were enriched by complementary use of TiO2 and Fe-NTA based affinity chromatography from digested lysates of macrophages from 4 healthy donors.

**Figure 4:**
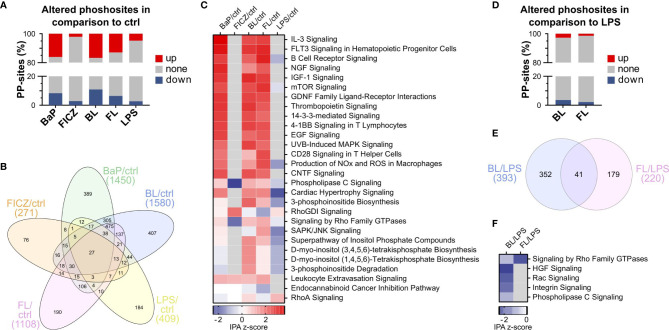
Effect of AhR-activation on protein phosphorylation. **(A)** Percentage of significantly altered PP-sites compared to DMSO (ctrl) after 2h stimulation. PP-sites were considered as significantly altered compared to the control with a p-value ≤ 0.01, as determined by Student’s t-test (n = 4). **(B)** Overlap of altered PP-sites between different treatments. **(C)** Pathways enriched among regulated PP-sites compared to the unstimulated control. Pathway enrichment analysis was conducted using the phosphorylation-dependent pathway analysis functionality of Ingenuity pathway analysis (IPA). Only pathways with an enrichment p-value ≤ 0.01 were considered enriched. Shown are the top 10 pathways enriched for each treatment. **(D)** Percentage of significantly altered PP-sites in BL- and FL-treated cells compared to LPS alone (p ≤ 0.01, n=4). **(E)** Overlap of altered PP-sites in BL and FL-treated macrophages. **(F)** Pathways enriched among PP-sites regulated after BL- or FL-treatment compared to LPS (p ≤ 0.01).

In total, 17,089 phosphorylation-sites (PP-sites) were identified. 9,742 PP-sites were reliably quantified in at least 3 of 4 replicates in one condition, with more than 5,400 sites for each condition (**Supplementary Table 3**). About 4,000 PP-sites were quantified in all six conditions. Significantly altered PP-sites were identified by Student’s t-test. The threshold for significant alteration was set at p ≤ 0.01 (**Supplementary Figure 6**). The most distinct alterations were found in BaP-treated samples (**Figure 4A**). BaP-treatment altered 24%, and BaP in combination with LPS-stimulation altered 26% of PP-sites compared to the unstimulated control. In contrast, FICZ-treatment and LPS-stimulation affected 5% and 7%, respectively. The combination of both stimuli altered 18% of PP-sites. All treatments, except FICZ, led to markedly more up-regulated than down-regulated PP-sites. A high overlap of 912 PP-sites was regulated by BaP and BL, while the two treatments showed only 389 and 407 unique PP-sites (**Figure 4B**). Only minor fractions were shared by only one of these treatments and FICZ, FL, or LPS. In contrast, the overlap in regulated PP-sites was less distinct for FICZ containing treatments. While FL affected four times more sites than FICZ, only 48% of FICZ-regulated PP-sits were shared with FL. BL mutually affected 69% of FL-regulated sites. However, LPS-treatment resulted in 51% of regulations being observed for at least BL or FL.

Next, signaling pathways affected by AhR-activation-dependent phosphorylations were identified using the phosphorylation pathway analysis function of Ingenuity Pathway Analysis (IPA). IPA identifies overrepresented pathways within differentially phosphorylated proteins and predicts pathway activity based on PP-site regulations (IPA z-scores). Pathways with an enrichment p-value ≤ 0.01 and absolute z-score ≥ 1 were considered as significantly altered. This approach identified 28 pathways significantly altered for at least one treatment (**Figure 4C**). Most of these pathways were activated (z-score ≥ 1) rather than deactivated (z-score ≤ -1). Interestingly, BaP, BL, and FL had a similar effect on the pathway level. Activation was most pronounced for IL-3 Signaling (z-score = 3.6) and FLT3 Signaling (z-score = 3.3) after BaP-treatment. Leukocyte Extravasation Signaling was the only pathway activated by all treatments with AhR-ligands (**Figure 4C**).

In contrast to the marked effects of BaP, BL, and FL, the effect of FICZ was limited (**Figure 4C**). Our data revealed that FICZ deactivated Phospholipase C Signaling and Signaling by Rho Family GTPases. In line with that, signaling by Rho GDP-dissociation inhibitor (RhoGDI Signaling), which negatively regulates Rho-family GTPases, was enhanced. Interestingly, LPS- treatment exhibited a deactivating effect on phosphorylation-dependent signaling after 2h of stimulation. None of the pathways activated after BL and FL treatment was enhanced after LPS-stimulation. RhoA Signaling was the only pathway that was activated by LPS (z-score = 1).

Comparing BL- and FL-stimulated with LPS-stimulated macrophages, 6% and 4% of PP-sites were regulated (**Figure 4D**). Up- and down-regulation appeared at nearly the same frequencies. The overlap of regulated sites for BL and FL was limited to 41 of 220 sites (**Figure 4E**).

Next, we determined AHR-induced phosphorylation-dependent signaling pathways altered in BL- or FL treated cells compared to LPS-stimulation (**Figure 4F**). Five pathways were affected by BL-treatment: HGF, Rac, Integrin, and Phospholipase C Signaling, and Signaling by Rho Family GTPases, which was the only pathway likewise affected by FL-treatment. All pathways were attenuated in double-stimulated macrophages. Our ubiquitome results showed increased ubiquitination of RAC1 after AhR activation, suggesting that this ubiquitination has mitigated Rac Signaling. Notably, Rac signaling cascades are also part of HGF and Integrin Signaling, indicating that attenuation of Rac Signaling can lead to the observed attenuation in HGF and Integrin Signaling.

Taken together, BaP, as well as double-stimulation with BL or FL, strongly regulated protein-phosphorylation, in contrast to FICZ or LPS. Enrichment analysis revealed that most pathways were activated after BaP-, BL-, or FL-treatment, whereas LPS had an attenuating effect on phosphorylation-dependent signaling. Comparing BL and FL to LPS-stimulation, all enriched pathways were attenuated. Most of these pathways depend on Rac or RhoGTPases, indicating a central role of these proteins for AhR-dependent effects.

### Altered Kinase Activities After AhR Activation

Next, we aimed to determine which kinases exhibit an altered activity after AhR activation and are thus likely responsible for the observed changes in protein phosphorylation. For this purpose, kinase activities were inferred by integrating amino acid sequence windows of significantly altered PP-sites with kinase-substrate motifs utilizing the KinSwingR package for the R environment for statistical computing (50), generating a normalized and weighted score for the predicted kinase activity (SWING). Additionally, the probability (p) for observation of other kinases with higher or lower SWING scores was calculated. Only kinases with p ≤ 0.05 were considered as significantly regulated.

**Figure 5:**
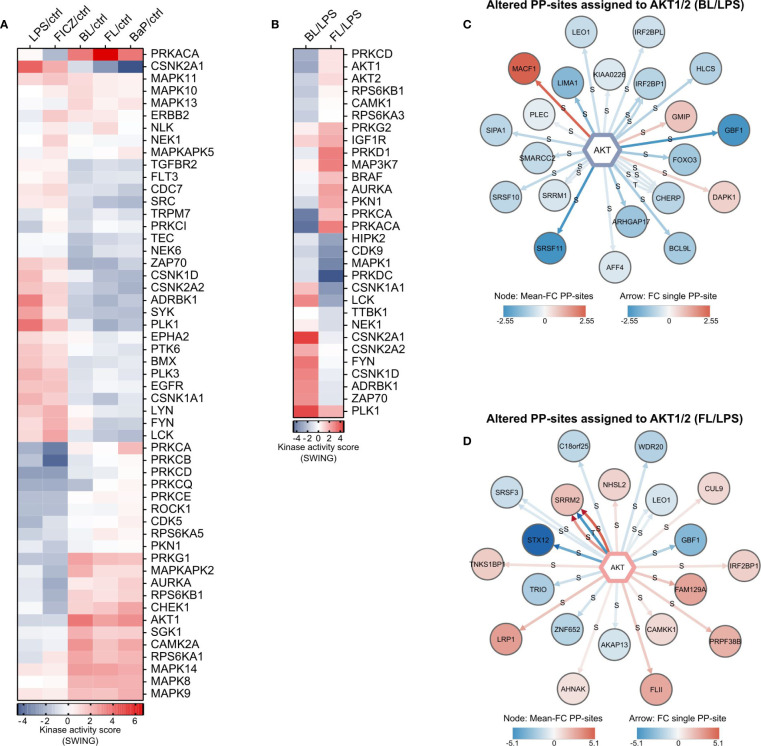
Inferred kinase activities and substrates. **(A)** Inferred alterations in kinase activities responsible for altered PP-sites in comparison to untreated cells, and **(B)** altered PP-sites in BL- or FL-treated macrophages compared to LPS-treated cells. Kinase-substrate motifs were calculated based on the kinase-substrate dataset from PhosphoSitePlus. Relative kinase activity scores (SWING) were calculated by integrating amino acid sequence windows of significantly altered PP-sites with kinase-substrate motifs utilizing KinSwingR. Altered phosphorylations in BL- **(C)** and FL- **(D)** treated cells compared to LPS-treatment assigned to AKT1/2. Data on regulated PP-sites assigned to AKT serine/threonine kinase (AKT) isoforms 1 and 2 were extracted after sequence-motive assignment. Each arrow represents a single PP-site. The color of the kinase target represents the mean FC over all assigned PP-sites.

In total, 53 kinases were found differentially active after at least one treatment compared to unstimulated control samples (**Figure 5A**). Thereby, LPS and FICZ-stimulated macrophages exhibited similar patterns of kinase activity. This extended to BaP-, BL-, and FL-stimulated cells and resembled the pattern observed for the regulation of signaling pathways (**Figure 4C**). Interestingly, SRC, which is part of the cytosolic AhR-complex (14), appeared to be deactivated after BL-, FL-, and especially BaP-treatment (SWING = -1.9). The most distinct activation was inferred for PRKACA after FL-treatment (SWING = 6.7). PRKACA was likewise activated by BL and BaP, although to a lesser degree. In contrast, PRKACA was less active after FICZ-treatment and unaffected by LPS alone. The most distinct deactivation was inferred for CSNK2A1 after BaP-treatment (SWING = -4.6). Noteworthy, treatment with LPS or FICZ resulted in the attenuation of protein kinase C (PKC) activity. Five PKC isoenzymes (PRKCA, PRKCB, PRKCD, PRKCQ, PRKCE) had reduced activity after both treatments. In contrast, all of these were unaffected by BL, FL, and BaP, with the exceptions of reduced PRKCQ activity after BL-treatment and slightly increased PRKCA activity after BaP-treatment.

Comparing BL- and FL-treated with LPS-treated cells, inferred activity was altered for 30 kinases (**Figure 5B**). Noticeably, regulations for most kinases were opposite for BL and FL. IGF1R and PRKG2 are the only kinases commonly more active after double-treatment compared to LPS alone. HIPL2, CDK9, MAPK1, and PRKDC are commonly less active, although the deactivation was markedly lower for BL.

Taken together, our ubiquitome and phosphorylation-dependent pathway analysis indicated alterations in RAC signaling. It was shown before, that signaling by RAC1 after TLR4 activation induces AKT serine/threonine kinase (AKT)-activity (63). Thus, changes in AKT-activity were further examined. The AKT isoforms AKT1 and AKT2 were both less active in BL-treated macrophages than after LPS-treatment (**Figure 5B**). Both kinases seemed activated after FL-treatment. However, the probability for kinases with higher SWING scores did not fulfill the significance threshold. 26 significantly altered PP-sites in 21 phosphoproteins were mapped to AKT1/2 after BL-treatment (**Figure 5C**). Of these sites, three were more abundant, while 23 PP-sites were down-regulated, indicating the reduced AKT activity after BL-treatment. Of the PP-sites with altered abundance after FL-treatment, 24 sites in 20 proteins were assigned to AKT1/2 (**Figure 5D**). Half of these were more, the other 12 were less abundant in FL-treated than in LPS-treated cells. Thus, AKT-activity was found decreased by BL-treatment and unaffected by FL-treatment compared to LPS-stimulation based on inference of kinase activity from the phosphoproteome data.

**Figure 6:**
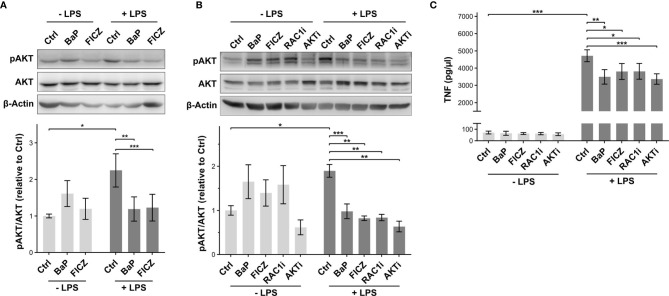
Effect of AhR activation on AKT phosphorylation. **(A)** Relative AKT phosphorylation levels at serine 473 (pAKT) were determined by western blot analysis 2h after stimulation. The pAKT signal intensities were normalized to total AKT levels. Shown is one representative blot and normalized intensities relative to LPS-treatment ± SEM (n = 7). Macrophages were additionally pretreated for 30min with the RAC1 inhibitor NSC23766 (RAC1i) and the AKT inhibitor Perifonsine (AKTi) before 2h incubation in the absence and presence of LPS. The relative levels of pAKT **(B)** and TNF-secretion **(C)** were measured by western blot and ELISA, respectively (n = 5). Significant changes were determined by two-sided, paired t-test (***p ≤ 0.001, **p ≤ 0.01, *p ≤ 0.05).

To validate the effect of BL on AKT-activity, the phosphorylation of AKT at serine 473 (pAKT) was analyzed by phosphosite specific western blot. The resulting intensities for pAKT were normalized to the total AKT signal (**Figure 6A**). As expected, LPS-treatment increased the pAKT level in macrophages. This induction was not found after co-treatment with BaP or FICZ, indicating a suppressive effect of the AhR-ligands on AKT activation. Next, the dependence of AKT phosphorylation on RAC1 activation was investigated utilizing a RAC1 specific inhibitor. Analysis of pAKT levels after RAC1-inhibition indicates that AKT-activity is diminished to a degree comparable to BL- and FL-treatment (**Figure 6B**). Direct inhibition of AKT led to a more distinct attenuation. The release of TNF was analyzed for the same set of samples (**Figure 6C**). Both, inhibition of RAC1 and AKT, led to a decrease in TNF-release after LPS-treatment comparable to the effect of the AhR-ligands BaP and FICZ.

The inference of kinase activity endorsed the ligand dependency of AhR induced regulations seen in the phosphoproteome data. At it, AKT, a downstream signaling component in RAC1-dependent TLR4 signaling, was attenuated after BL-treatment compared to LPS. The AKT-activating Rac signaling was attenuated by BL, as observed in our phosphoproteome. Inhibition of RAC1 reduced AKT phosphorylation in response to LPS-treatment. The reduced AKT phosphorylation was associated with a reduction of TNF-release.

## Discussion

The AhR has important immunomodulatory properties, such as limiting the release of pro-inflammatory cytokines and protection from septic shocks, and can signal through genomic and non-genomic mechanisms (2, 9, 10, 19). Likewise, a decrease in the release of pro-inflammatory TNF was observed in this study and previously described (25). However, molecular mechanisms are not fully understood. Using a multi-PTM-omics approach, we unraveled the mechanisms of non-genomic AhR-signaling in immunomodulation. The alterations of protein abundance, ubiquitination, and phosphorylation after treatment with the endogenous AhR-ligand FICZ and exogenous ligand BaP were analyzed in endotoxin-stimulated human monocyte-derived macrophages. Furthermore, kinase-activities were inferred from our phosphoproteome results.

### Limited Response by Altered Protein Abundance

The effect of AhR activation on protein abundance observed in this study was minute. Treatment with AhR-ligands (BaP or FICZ) alone led to no commonly regulated proteins, while five were commonly regulated after co-stimulation with LPS. Such a limited response to BaP after short incubation time (2h) was previously described for the murine hepatoma cell line Hepa1c1c7, where alterations in protein levels peaked after 4h and 24h (55). To our knowledge, no transcriptome dataset matching cell type and stimulation used in this study is available jet. Though, Sparfel et al. demonstrated that, after 8h of BaP-exposure, 96 genes are differentially expressed in human macrophages, while 1100 genes are affected after 24h (64). Together, these data suggest that AhR-dependent alteration in protein level has a later onset than 2h after ligand-treatment, indicating regulations of protein ubiquitination and phosphorylation depend rather on non-genomic mechanisms than induced protein expression by the transcriptional activity of AhR.

### Immunomodulation on the TLR4-RAC1-PI3K-AKT Axis

Regulation of protein activity through modifications like ubiquitination and phosphorylation is typically faster than regulation through protein abundance. In addition, PTM-dependent signaling cascades are often responsible for altered gene expression. Signaling of LPS-triggered TLR4 *via* the canonical MyD88-dependent pathway essentially depends on the ubiquitination of multiple proteins (61). All ubiquitinations involved in MyD88-dependent TLR4-signaling identified in this study were increased after LPS-stimulation, indicating a sustained TLR4-activation. AhR activation by BaP or FICZ did not affect these ubiquitinations. Merely, the ubiquitination of IκBα was slightly increased after double-stimulation. Hence, non-genomic AhR-signaling seems not to modulate MyD88-dependent TLR4-activation through altered ubiquitination.

Consequently, we focused on ubiquitinations mutually regulated by both AhR-ligands to identify Ub-dependent signaling events modulated directly by AhR acting as an integral component of an E3 ubiquitin ligase complex or indirectly after AhR activation. Most of the 36 Ub-sites induced by AhR activation were enhanced, antagonized, or induced to a similar degree by LPS-treatment. Only two Ub-sites, RAC1_147 and RAP1A_73, were up-regulated by BaP and FICZ completely independent of LPS-treatment. Besides interfering signaling cascades, an explanation for the antagonization of AhR-dependent ubiquitination by LPS relies on the strong induction of protein ubiquitination by LPS. A strong increase of poly-ubiquitinations leads to limitations in the pool of free ubiquitin, which results in a deprivation of Ub from other ubiquitinations, especially mono-ubiquitinations (65–67).

Interestingly, the ubiquitination of RAP1A at Lysine 73 was previously not described and not listed in the PhosphoSitePlus or BioGRID database (51, 68). RAP1A protein levels did not decrease after BaP or FICZ-treatment, indicating non-degradative ubiquitination. Shao et al. reported a negative regulation RAP1A-activity through an E3 ubiquitin-protein ligase CBL (CBL)-dependent, but proteolysis-independent mechanism (69). AhR activation by tetrandrine activates CBL in an SRC-dependent, non-genomic manner. This activation leads to ubiquitination and degradation of Tyrosine-protein kinase SYK (22). The SRC-activity inferred from our phosphoproteome did not increase after AhR activation. However, phosphoproteome and ubiquitome were analyzed after 2h treatment, whereas SRC-activation is an initial event after AhR activation. The initially increased SRC-activity decreases by 50 % within the first 25 min after AhR-ligand administration (70). In macrophages, RAP1A is required for Fc gamma receptor-dependent phagocytosis (71). Additionally, RAP1A activation fosters superoxide generation during phagocytosis of IgG-opsonized zymonans, mechanistically involving the release of RAC1 from the RAC1-RhoGDI complex (72).

The ubiquitination of RAC1 at its major ubiquitination site K147, which is the Ub-site regulated in this study, is dependent on the activity of c-Jun N-terminal kinases (MAPK8, MAPK9, MAPK10) (73). The kinase activity of MAPK8 and MAPK9 was markedly increased by BaP-treatment and double-stimulation with LPS and AhR-ligand. The activity increase was smaller for FICZ. The same was observed for MAPK10, although to a lesser extent. The lower increase of MAPK8/9/10-activity after FICZ-stimulation is in line with a less distinct up-regulation of the observed RAC1-ubiquitination. Therefore, the increased RAC1_K147-ubiquitination is likely dependent on AhR-induced activity of the c-Jun N-terminal kinases JNK. RAC1 links TLR-activation to the PI3K-Akt signaling pathway contributing to pro-inflammatory signaling in macrophages after LPS-encounter (74, 75). The increased ubiquitination potentially interferes with this activation pathway, which is additionally supported by further components of PI3K-AKT-signaling (LAMA1, MCL1, YWHAB, BCL2L1) being ubiquitinated after AhR activation. In line, Rac Signaling was found to be decreased in BL-stimulated macrophages compared to LPS-activation alone.

These cells likewise showed a decrease in AKT-activity based on kinase activity inference from the phosphoproteome data. The decreased AKT-activity could be confirmed by western blot analysis of the activation-dependent AKT phosphorylation at serine 473. Differing from the prediction by kinase activity inference, AKT activity was found attenuated for BL and FL compared to LPS. The kinase activity inference is based on sequence windows of significantly altered PP-sites mapped to kinase-substrate motifs (50). Likely, inference of kinase activity was more precise due to the broader data basis of regulated phosphorylation sites for BL than FL (393 versus 220). Utilizing a RAC1-specific inhibitor (76), the decreased pAKT-levels in BL- and FL-treated cells were mimicked in this study, confirming that a decreased RAC1-activity leads to decreased AKT-activity in LPS-treated macrophages. Inhibition of RAC1 or AKT led to an attenuated TNF-release after LPS-stimulation similar to the co-treatment with the AhR-ligands BaP or FICZ. In sum, these findings indicate that AhR-activation attenuates the TLR4-RAC1-PI3K-AKT axis.

In addition to the connection of TLR4-activation to the PI3K-Akt signaling pathway, RAC1 has been shown to link LPS-stimulation and the formation of reactive oxygen species (ROS), which stimulates NF-κB activation and subsequent TNF-secretion (74). In this process, RAC1 is required for the recruitment of the NADPH oxidase activating subunit neutrophil cytosol factor 2 (NCF2 or p67^phox^) to the membrane of phagocytes (77) and to keep NCF2 in the active conformation (78). Based on our data, it is not possible to exclude an effect of BaP or FICZ on the RAC1-dependent activation of NADPH oxidases. Nevertheless, the finding that inhibition of AKT led to a decrease in TNF-secretion comparable to the effect of the AhR-ligands is an indicator for a subsidiary role of an altered RAC1-dependent NADPH oxidase activity at the investigated time point and ligand concentrations.

Besides providing a second route for IκBα-phosphorylation after TLR4-activation, AKT phosphorylates additional targets like BAD, TSC2, and CREB, thereby directly impacting macrophage survival, polarization, and autophagy (79, 80). One of these additional targets is Forkhead box protein O3 (FOXO3), which was found less phosphorylated in BaP- and BL-treated cells. The result of this phosphorylation is the inhibition of the transcription factor FOXO3 (81), suggesting an enhanced activity of FOXO3 after AhR activation by BaP. The inhibition of FOXO3 after TLR4 activation typically results in suppressed autophagy (82). In addition, AKT-dependent inhibition of FOXO3 through phosphorylation enhances the production of the anti-inflammatory cytokine IL-10 (83). Therefore, decreased FOXO3-phosphorylation would suppress IL-10 levels. In contrast, AhR activation is thought to increase IL-10-expression through SRC-dependent signaling (26). The balance of these two competing pathways may have an important role in AhR immunomodulatory functions.

**Figure 7:**
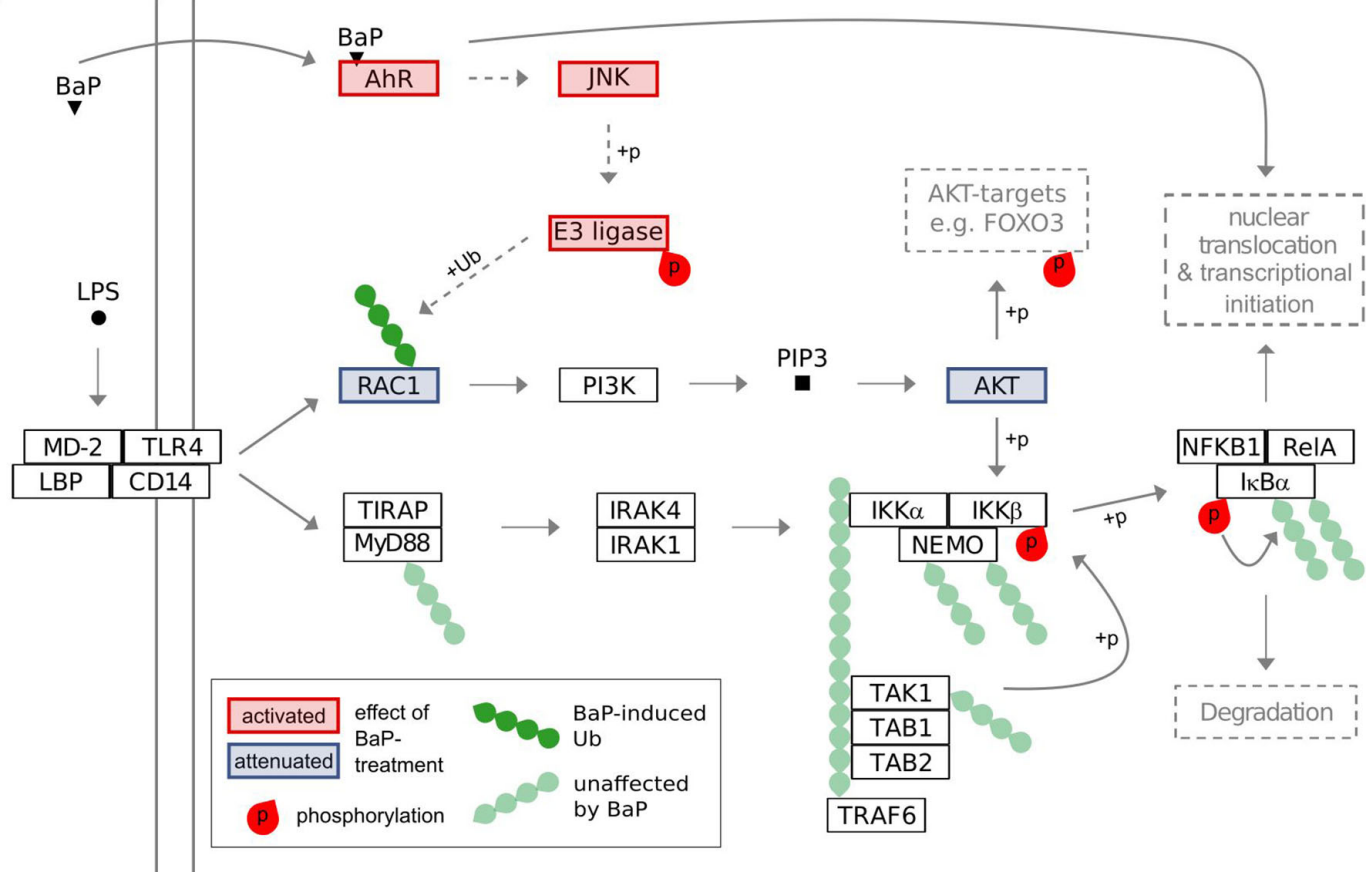
Suggested model of interference of BaP-activated AhR with TLR4 signaling. The model is based on the results of the conducted multi-PTM-omics study and the KEGG-pathways for Toll-like receptor signaling and PI3K-Akt signaling.

Taken together, our data suggest that AhR activation by BaP induces JNK-dependent RAC1-ubiquitination, which inhibits the RAC1-PI3K-AKT-signaling axis after TLR4-activation and thus potentially contributes to the AhR-dependent attenuation of pro-inflammatory signaling in a non-genomic manner (**Figure 7**).

### The Non-Genomic Immunomodulatory Response to AhR Activation Is Ligand-Dependent

The inhibitory effects of AhR-stimulation on Rac Signaling and AKT-activity in our phosphoproteome dataset were mainly observed for BaP-treatment. In contrast, FICZ did not alter Rac signaling but showed a comparably decreased AKT phosphorylation in western blot analysis. Although the used concentrations of BaP and FICZ led to comparable genomic responses as demonstrated by similar up-regulation of CYP1B1 and AHRR mRNA-expression, the non-genomic consequences differed. About 5-times more Ub- and PP-sites were regulated after BaP-treatment than after FICZ-administration. However, the differences diminished in macrophages treated with BaP or FICZ combined with LPS. Especially phosphorylation-dependent signaling and kinase-activities compared to LPS-treated macrophages were still divergent.

Different responses by the immune system to different AhR-ligands have been previously reported. TCDD induces differentiation of functional regulatory T cells, which suppress experimental autoimmune encephalomyelitis, whereas FICZ leads to T_H_17 cell differentiation, which worsen disease progression in the same model system (84). Prolonged AhR activation with FICZ does not affect neutrophil recruitment or inducible nitric oxide synthase levels in a mouse model of influenza virus infection. In contrast, both are augmented by TCDD-treatment (85). It is suggested that the duration of AhR activation is a primary factor of AhR-ligand effects on the immune system (86). While TCDD is persistent with long *in vivo* half-life, FICZ is rapidly metabolized, requiring AhR-dependently expressed CYP1A1 and CYP1B1 (87, 88). After 5h, nearly no FICZ remains after the treatment of HepG2 cells (89). BaP, the exogenous ligand used in this study, is degraded likewise and metabolized nearly completely 24h after administration (55).

The induction of the metabolizing enzymes was not yet observable after 2h treatment, which was used in this study. Nevertheless, the induction was clear on the mRNA level after 6h, indicating that ligand-degradation did not take part at the analyzed time-point. More likely, the structure of the ligands influences non-genomic AhR-dependent immunomodulation. Such a relation is known for other ligand-activated nuclear receptors, as reviewed by Jin and Li (90). The binding of structurally differing ligands results in different conformational states of the receptor, which then favors the binding of other proteins (90). Furthermore, a relation between structural ligand-properties and the results of AhR activation was previously described (91). Differences in ligand-binding-properties are sufficient for developing AhR-ligand-specific antagonists like the TCDD-antagonist CH223191 (92).

Both the higher persistence and different conformational changes after BaP-binding to AhR potentially contribute to the BaP-specific effects on protein ubiquitination and phosphorylation. However, through targeting AKT-activity, the effects of BaP and FICZ converged regardless of the differences in the phosphoproteome analysis. Finally, the mechanisms underlying the observed ligand-specific responses are not clear yet and need further investigations. In this study, FICZ, a UV light-dependent Tryptophan product, was investigated as endogenous AhR-ligand. Further Tryptophan-metabolites have been shown to activate or antagonize AhR, with many being derived from the intestinal microbiota (93). At the origin of these ligands, AhR is essential for intestinal immunity and barrier function (94, 95). Therefore, further endogenous ligands may be taken into account to better understand AhR-functions under physiological conditions and diseases. Moreover, considering the BaP-specific effects and the attenuation of LPS-dependent macrophage activation, BaP might act as a modulator of the microbiome-host interaction.

### Conclusions

In this study, the non-genomic AhR-signaling and its role in immunomodulation were investigated. Therefore, alterations in protein abundance, ubiquitination, and phosphorylation were globally addressed utilizing respective omics approaches before the visibility of genomic signaling on the protein level. The observed alterations in post-translational modifications exhibited a dependency on the administered AhR-ligand. Finally, inhibition of the RAC1-PI3K-AKT signaling-axis in TLR4-signaling after BaP- or FICZ administration was insinuated. The inhibitory mechanism involves AhR-dependent JNK-activity and subsequent RAC1-ubiquitination. Conclusive, the obtained data may prove a valuable resource for further elucidation of non-genomic AhR-signaling in immunomodulation and their potential for targeting in AhR-dependent pathologies, e.g., atherosclerosis and major depressive disorder or diseases associated to a disturbed intestinal immunity.

## Data Availability Statement

The mass spectrometry proteomic data presented in the study are publicly available. This data can be found here: ProteomeXchange Consortium (http://proteomecentral.proteomexchange.org) via the PRIDE partner repository (96) with the dataset identifier PXD020770.

## Ethics Statement

The studies involving human participants were reviewed and approved by Ethics Committee of the University of Leipzig (Ref. #079-15-09032015). The patients/participants provided their written informed consent to participate in this study.

## Author Contributions

Conceptualization: MB and KS. Methodology: HG and KS. Software: IK and HG. Investigation: HG and KW. Writing—original draft: HG and KS. Writing—review and editing: HG, KW, IK, MB, and KS. All authors contributed to the article and approved the submitted version.

## Funding

This work was supported by the Helmholtz Association of German Research Centers. This study was supported by a grant of the Deutsche Forschungsgemeinschaft (DFG) TRR67Z4 to MB and HG.

## Conflict of Interest

The authors declare that the research was conducted in the absence of any commercial or financial relationships that could be construed as a potential conflict of interest.
