# Supplementary material for: Non-Genomic AhR-Signaling Modulates the Immune Response in Endotoxin-Activated Macrophages After Activation by the Environmental Stressor BaP
Source: Front Immunol. 2021 Mar 31;12:620270. doi: 10.3389/fimmu.2021.620270 (PMC8045971; doi:10.3389/fimmu.2021.620270)
Supplement: Supplementary file 4 [file DataSheet_1.docx]

Supplementary Material

# Supplementary Data Tables

Supplementary Table 1: Quantified proteins and results of statistical analysis

Supplementary Table 2: Relative abundance of quantified Ub-sites

Supplementary Table 3: Quantified PP-sites and results of statistical analysis

# Supplementary Figures

**
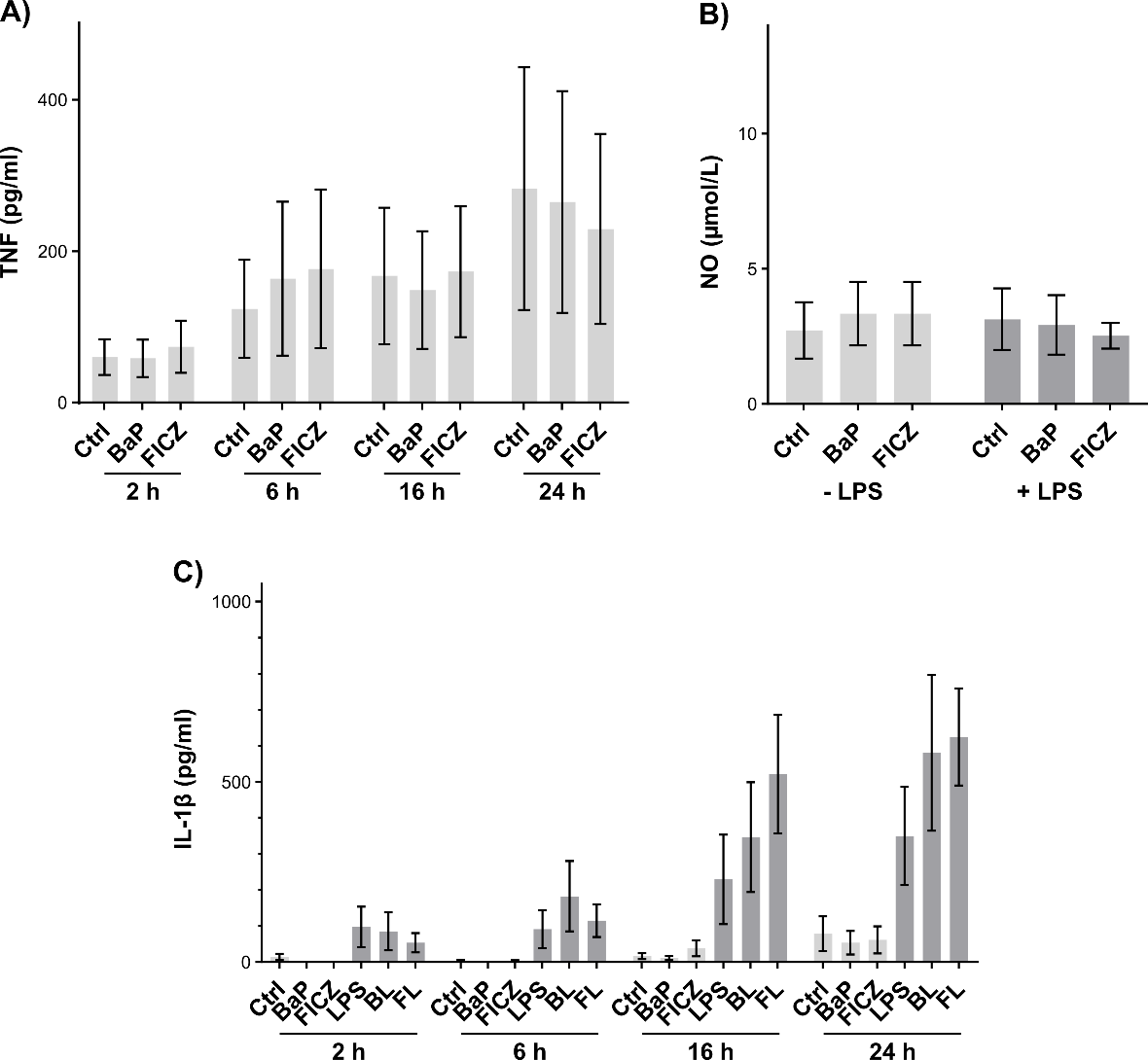
**

**Supplementary Figure 1: Secretion of TNF, nitric oxide (NO), and IL1β.** **(A)** The release of TNF to the cell culture supernatant was measured 2h, 6h, 16h, and 24h after treatment with BaP (2µM) or FICZ (100nM) by ELISA. Data are shown as mean ±SEM (n = 5). **(B)** The release of nitric oxides (NO) in response to BaP (2 µM) and FICZ (100 nM) in the absence and presence of LPS (100 ng/ml) was determined by colorimetric assay 2h post stimulation (n=4). **(C)** The secretion of IL1β was measured 2h, 6h, 16h, and 24h after treatment with BaP (2µM) or FICZ (100nM) in the absence and presence of LPS (100 ng/ml) by ELISA. Data are shown as mean ±SEM (n = 4). No significant changes were determined by two-sided, paired t-test.


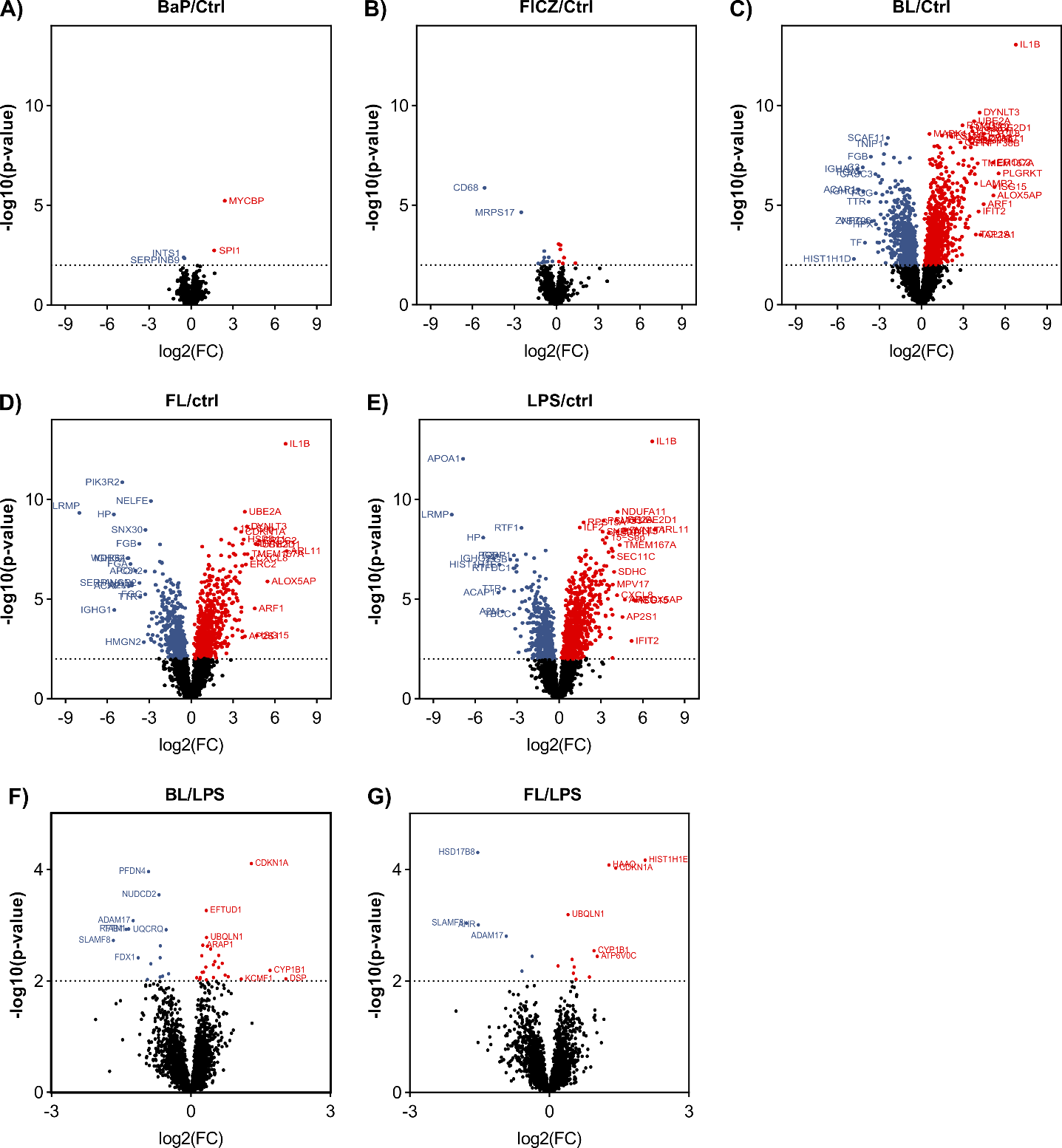


**Supplementary Figure 2: Identification of significantly regulated proteins.** (A-E) Volcano plots showing log2(FC) and –log10(p-value) comparing BaP-, FICZ-, BL-, FL-, or LPS-treatment with the unstimulated control and (F-G) comparing BL or FL with LPS-treatment. Each dot represents a protein. Significantly up-regulated proteins are shown in red and down-regulated proteins are shown in blue. The threshold for significant regulation was p ≤ 0.01 (dotted line). Protein p‑values were determined by two-sided, unpaired t-tests (n = 7).


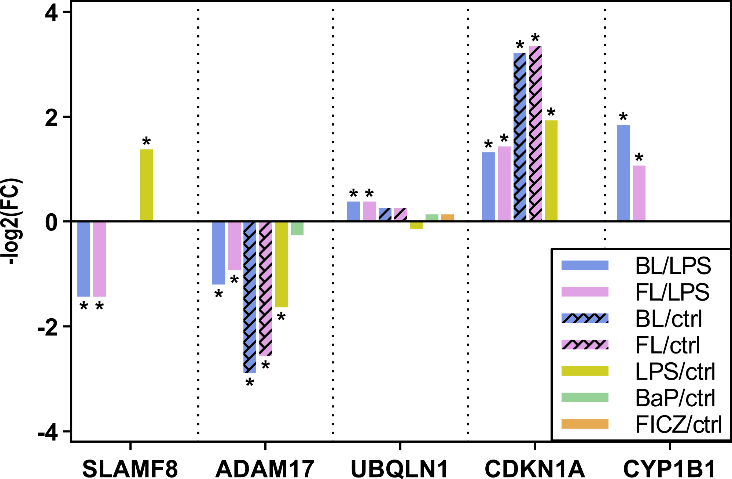


**Supplementary Figure 3: Proteins commonly regulated after BL- and FL-treatment compared to LPS-stimulation.** Shown are the mean log2(FC). (* = p ≤ 0.01, two-sided t-test, n = 7).


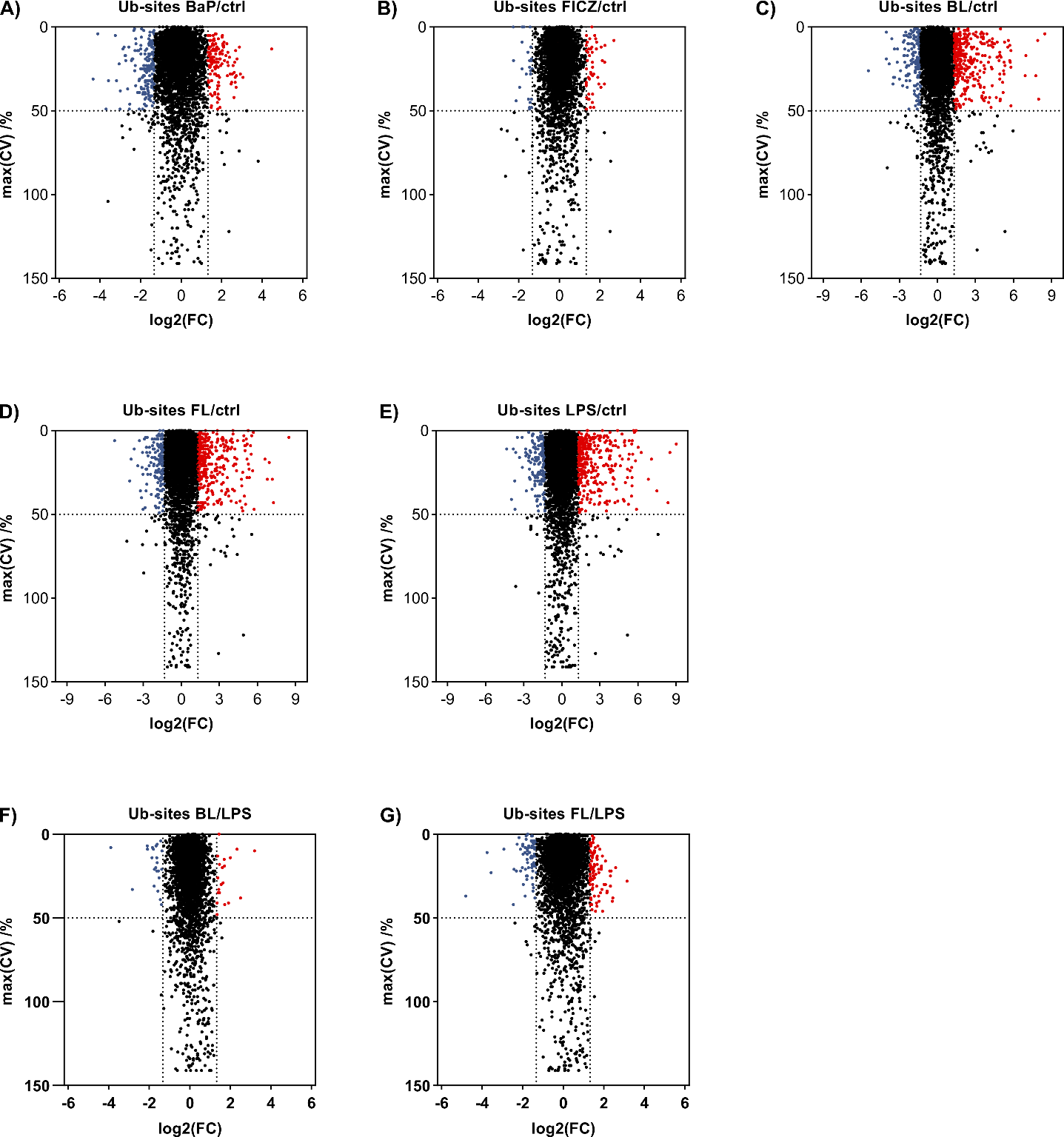


**Supplementary Figure 4: Identification of altered Ub-sites.** (A-E) Maximum coefficient of variation (max(CV)) in dependency of the Ub-site log2(FC) comparing BaP-, FICZ-, BL-, FL-, or LPS-treatment with the unstimulated control and (F-G) comparing BL or FL with LPS-treatment. Each dot represents an Ub-site. More abundant Ub-sites are shown in red and less abundant Ub-sites are shown in blue. Pools from nine healthy donors were measured in duplicates for each condition. Ub-sites with an absolute FC ≥ 2.5 and a max(CV) ≤ 50 % were considered as altered.


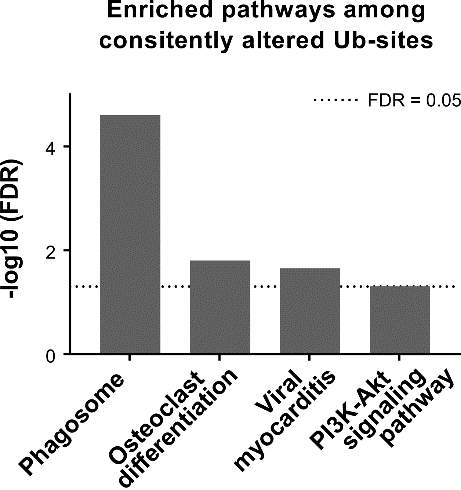


**Supplementary Figure 5: KEGG-pathways enriched among mutually altered ubiquitinated proteins.** Pathway enrichment analysis for proteins mutually altered ubiquitinated after BaP- and FICZ-treatment were conducted using the DAVID bioinformatics resources against the KEGG reference database. Shown are all pathways with an FDR ≤ 0.05.


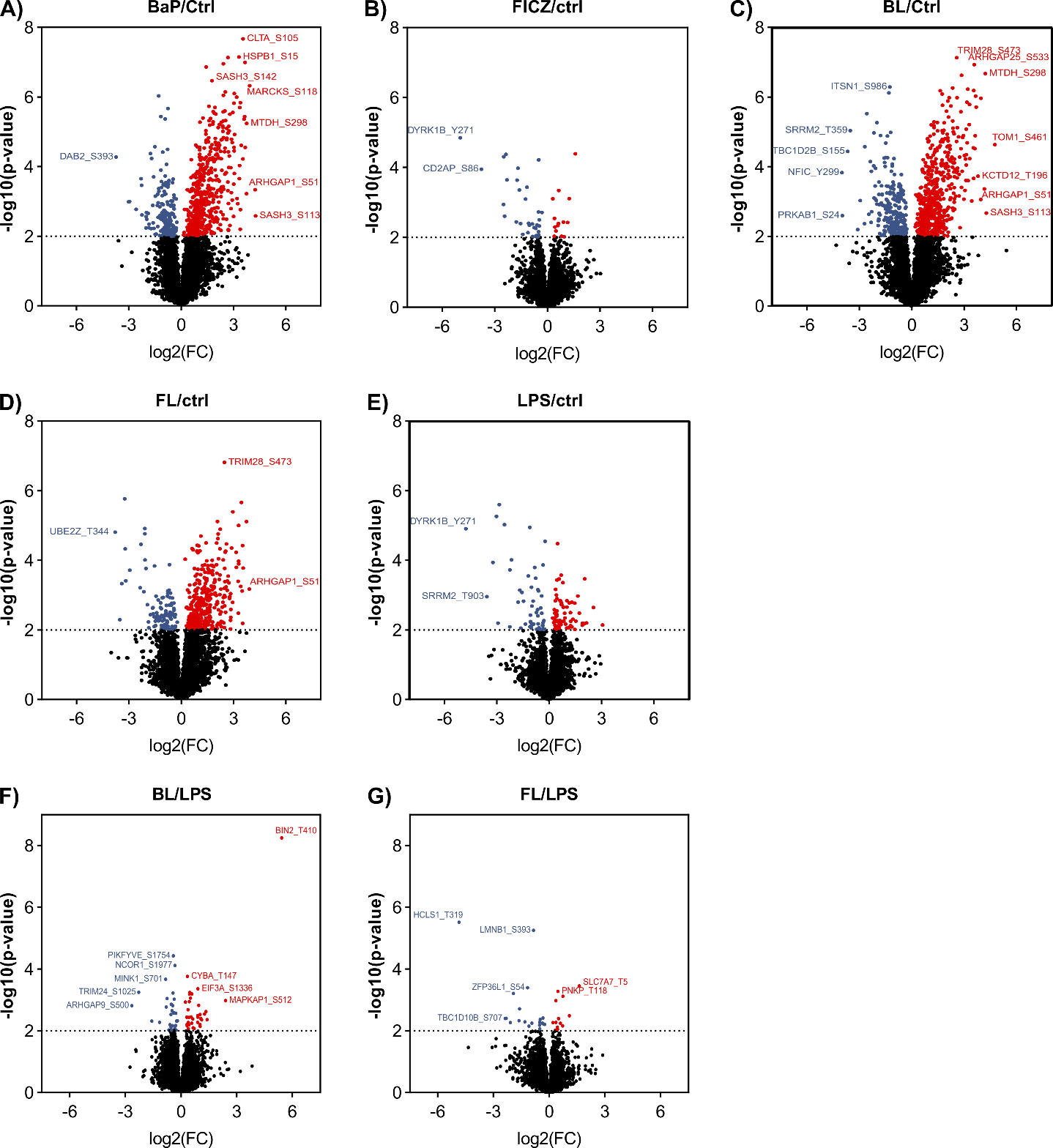


**Supplementary Figure 6: Identification of significantly regulated phosphosites.** (A-E) Volcano plots showing log2(FC) and –log10(p-value) comparing BaP-, FICZ-, BL-, FL-, or LPS-treatment with the unstimulated control and (F-G) comparing BL or FL with LPS-treatment. Each dot represents a phosphosite. Significantly up-regulated phosphosites are shown in red and down-regulated phosphosites are shown in blue. The threshold for significant regulation was p ≤ 0.01 (dotted line). PP-site p‑values were determined by two-sided, unpaired t-tests (n = 4).
